# Objective Accuracy in Estimating Repetitions in Reserve in the Back Squat: An Analysis between Experienced vs. Novice Subjects

**DOI:** 10.5114/jhk/205218

**Published:** 2025-11-19

**Authors:** Felipe Andrés Bermúdez Droguett, Raúl Ricardo Festa, Naomy Alexandra Telchi Quintana, José Gomez-Lopez, Álvaro Huerta Ojeda, Claudio Farias-Valenzuela, Frano Giakoni-Ramírez, Emilio Jofré-Saldía

**Affiliations:** 1 Motion Human Performance Laboratory, Santiago, Chile.; 2 Department of Exercise Science, Sports Performance Research, Rosario, Santa Fe, Argentina.; 3 Facultad de Filosofía y Humanidades, Universidad Austral de Chile, Valdivia, Chile.; 4 Núcleo de Investigación en Salud, Actividad Física y Deporte ISAFYD, Universidad de Las Américas, Viña del Mar, Chile.; 5 Escuela de Ciencias de la Actividad Física, el Deporte y la Salud, Universidad de Santiago de Chile (USACH), Santiago, Chile.; 6 Facultad de Educación y Ciencias Sociales, Universidad Andres Bello, Santiago, Chile.; 7 Escuela de Ciencias de la Actividad Física, Facultad de Ciencias de la Rehabilitación y Calidad de Vida, Universidad San Sebastián, Santiago, Chile.

**Keywords:** resistance training, training close to failure, perceived effort, movement velocity, training experience

## Abstract

This study aimed to objectively compare the accuracy of estimating repetitions in reserve (RIR) in the back squat among subjects with different levels of experience in resistance training (RT). Sixteen healthy adults (24.31 ± 4.94 years, 4 women and 12 men) were divided into the experienced (N = 8; ≥ 18 months of experience) and the novice (N = 8; <18 months of experience) in RT. Each group performed one set to muscle failure (day 1), and one RIR3 and RIR1 (day 2) of Smith machine back squats with a load of 70% 1RM. In addition, mean propulsive velocity (MPV) and the rating of perceived exertion (RPE; 0–10 scale) were assessed to individually objectify the RIR. There were no differences in accuracy for estimating both RIR3 and RIR1 between groups (p > 0.05, d ≥ 0.03). Furthermore, there were no differences in MPV, velocity loss, the RPE, and total of repetitions to muscle failure, RIR3, and RIR1 sets (p > 0.05, d ≥ −0.07). Overall, our findings suggest that RT experience does not influence the accuracy of estimating RIRs in the back squat, which is objectively supported by individualized MPV and the RPE. Previous familiarity with high-level effort may play a key role in accuracy beyond RT experience.

## Introduction

Resistance training (RT) is widely recognized for its effectiveness in increasing muscle mass and strength, benefiting health, preventing disease and improving athletic performance (Damas et al., 2019). In this context, momentary muscle failure is defined as "the inability to complete the concentric phase of a repetition despite maximum effort to do so" (Robinson et al., 2024) where the proximity is considered a key factor in RT (Refalo et al., 2022), and related to the level of effort applied (Hernández-Belmonte et al., 2022; Sánchez-Medina and González-Badillo, 2011). In this regard, the level of effort is represented by the percentage or the number of repetitions performed with respect to the maximum possible and associated with the velocity loss (VL) during a set (Bachero-Mena et al., 2025; Sánchez-Moreno et al., 2021), which potentially addresses the neuromuscular adaptations of a RT program (Pareja-Blanco et al., 2020).

A recent review suggests the use of simple and practical tools to regulate the level of effort during RT in the general population, such as the rating of perceived exertion (RPE) and repetitions in reserve (RIR) (Festa et al., 2023). RIR are defined as the number of repetitions that an individual estimates to be able to perform until reaching muscle failure (Jukic et al., 2024). However, despite its wide application, this tool depends on the individual subjective accuracy. In this sense, previous studies have shown that the perception of the ability to continue performing repetitions to failure can vary among individuals with different levels of RT experience (Steele et al., 2017; Zourdos et al., 2016). Nevertheless, other studies have found opposite results, where training experience did not affect the accuracy of RIR estimation (Hackett et al., 2017; Halperin et al., 2022; Refalo et al., 2024; Remmert et al., 2023). In this regard, a recent review points out that RT experience and RIR are areas for future research to clarify (Bastos et al., 2024).

To date, it is important to highlight that studies of the RIR accuracy in individuals with different levels of experience have some particularities in the applied methodology that increase the level of subjectivity. Specifically, in some research participants are asked before (Steele et al., 2017) or during (Refalo et al., 2024; Remmert et al., 2023) the set about the number of repetitions that they estimate they can perform to failure, and then continue the repetitions until failure, comparing the difference between their prediction and the completed repetitions. One of the disadvantages of these methodologies is that they generally lack an objective approach to estimating RIR, particularly when comparing individuals with different levels of experience. In this sense, Odgers et al. (2021) related the rating of perceived exertion (RPE) to RIR, and the RPE to movement velocity during sets to failure in subjects with at least 6 months of RT experience. Although those authors indicated a good relationship between the RPE with RIR and movement velocity supporting the use of RIR (Odgers et al., 2021), this was not directly linked to movement velocity, which could have objectified its application through a valid tool. Furthermore, this approach was also applied to compare subjects with different levels of experience, showing similar results (Zourdos et al., 2016). For more objective support, it has recently been observed that movement velocity can accurately and objectively estimate the level of effort based on RIR, regardless of the level of experience (Morán-Navarro et al., 2019); however, we are aware that measuring movement velocity is not accessible to everyone (e.g., cost of the devices), thus it is essential to integrate it with more accessible tools. Although there is evidence highlighting the objectivity of RIR, to our knowledge no study has directly analyzed its relationship with valid (i.e., movement velocity) and practical (i.e., RPE) tools to compare the accuracy of estimations in RIR among subjects with different levels of experience in RT more objectively.

Therefore, the purpose of the present study was to compare the accuracy in RIR estimation in the back squat among subjects with different levels of experience, objectifying the accuracy with movement velocity and the RPE. This study attempted to address inconsistencies in previous research on RIR accuracy according to the RT experience level (Refalo et al., 2024; Remmert et al., 2023; Steele et al., 2017; Zourdos et al., 2016), and to provide greater objectivity in accuracy by incorporating valid and practical tools.

## Methods

### Participants

Sixteen healthy adults (24.31 ± 4.94 years; 77.59 ± 14.92 kg; 172.00 ± 8.81 cm; 4 women and 12 men) attending a training center were divided according to their RT experience, [a] ≥ 18 months of experience (Experienced Group; EG: 2 women and 6 men), and [b] <18 months of experience (Novice Group; NG: 2 women and 6 men). Participants were not undergoing any training program at the time of the study other than their usual RT. For women, their menstrual periods were not monitored or recorded. The 18-month criterion for classifying experience was similar to and supported by previous research (Zourdos et al., 2016), and that was a time that ensured the transition from an initial stage of learning to a consolidation phase in training (Steele et al., 2017). Inclusion criteria were (i) healthy men and women aged 18–35 years, and (ii) use of some squat variant (free weight or Smith machine) in their current RT program. Exclusion criteria were: (i) lower limb injuries in the last 6 months, (ii) illness or disease, (iii) use of ergogenic supplements and/or medication. The sample size for this research involved a selection procedure guided by the characteristics and purposes of the research, which is why we referred to a non-probability and convenience sample since it was available during the time and the period of the research (Vehovar et al., 2016). All participants gave written informed consent to take part in the intervention. The study’s protocol was approved by the Ethics Committee of the Universidad de Las Américas, Chile (registration number: CEC_FP_2023011). The present study was conducted in accordance with the ethical principles of the Declaration of Helsinki and the Ethical Standards in Sport and Exercise Science Research (Harriss et al., 2019).

### Sample Size Justification

This factor had two practical considerations: [1] recruiting more than 16 subjects was not possible due to time and location availability related to data collection; [2] although previous studies on RIR had a larger number of subjects (Refalo et al., 2024; Zourdos et al., 2016), they did not perform a comprehensive comparison of variables to determine the influence of experience on RT. However, since the a priori sample size calculation was not performed, a post hoc power analysis was conducted using G*Power software (Version 3.1.9.6; Faul et al., 2007) to justify the validity of the present study based on the magnitude of the effects detected. Two-tailed analysis for two independent means yielded a large effect size (*d* > 0.80) for a probability of α = 0.05 (type I error) with a power = 0.80 (1-β err prob; type II error) for a total of 16 subjects (8 per group). Although the sample size was small, these results indicate that the study was adequately powered to detect large effects (i.e., relevant to practice). However, we acknowledge that the sample size may limit the generalizability of the findings.

### Design and Procedures

At the first visit, participants read and signed informed consent and underwent anthropometric assessments (SECA 213^®^ measuring rod; OMRON HBF-514^®^ electric bioimpedance scale). After exercise familiarization, all participants performed two sets of 8 to 12 repetitions of the back squat on a Smith machine (Generic Brand, China) with no added load, which was used for all interventions in this study. Consistent technique was encouraged throughout all repetitions, reaching at least 90 degrees of knee flexion, with no change in range of motion or technique as the level of effort increased. After individual adjustments, the floor was marked to maintain the execution position in a standardized manner. To determine the external load, four sets of 1 to 3 repetitions were performed with progressive loads between ~50% and ~90% of one repetition maximum (1RM) according to weights and repetitions reported by participants and then estimated with the Brzycki's formula (Brzycki, 1993) with passive rest periods of 3 min between each set (Grgic et al., 2018; Rosa et al., 2023), where the mean propulsive velocity (MPV) was measured at each repetition using Valkyria Trainer Free encoder^®^ (Ivolution; Sunchales, Argentina). This device is valid and reliable for such measurements since it presents high agreement with gold standard devices like ChronoJump^®^ (coefficient of variation <8% and intra-class correlation coefficient [model 3.1] >93%; Huerta Ojeda, *unpublished data*). Participants were instructed to perform the concentric phase of the back squat at maximal intended velocity (Sánchez-Medina et al., 2017). This protocol allowed estimating the current 1RM of each participant using the linear encoder software (Valkyria Trainer Free encoder^®^ by Ivolution; Sunchales, Argentina). Subsequently and after a period of at least 5 min of passive rest, each participant performed a single set until momentary muscle failure, that is, when the participant was not able to complete the concentric phase of a repetition despite the maximum effort to do so (Robinson et al., 2024), to determine the individual velocity-RIR profile (Balsalobre-Fernández et al., 2021a) in the back squat exercise with an external load of 70% 1RM, according to the 1RM estimated using the linear encoder software. Unlike the load-velocity assessment, in this protocol participants were instructed to perform the eccentric and concentric phases of each repetition at their own speed with no more than 5 s between repetitions until momentary muscle failure, with the aim of improving ecological validity (Odgers et al., 2021). Participants were encouraged to maintain the same technique as in the previous test. At the end of the series until momentary muscle failure, the perception of effort was measured (10-point facial RPE scale [FCR10]; van der Zwaard et al., 2023).

**Figure 1:**
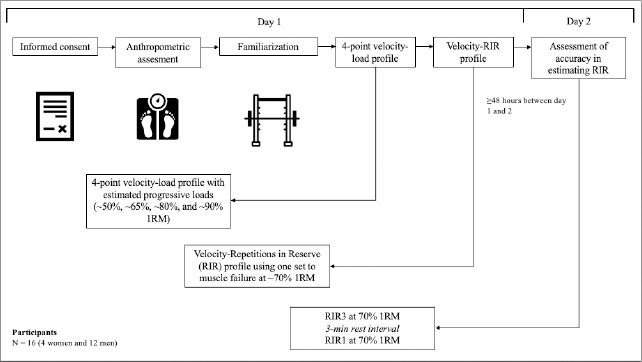
Summary of the study design and the intervention.

During the second visit, after familiarization with the concept of RIR, participants performed two sets of the back squat exercise with the same external load as the set to muscle failure of the first visit (i.e., 70% 1RM) with 3 min of passive rest and the same ecological validity criteria as aforementioned, voluntarily ending each set when they considered themselves to be 3 and 1 repetitions to failure (i.e., RIR3 and RIR1, respectively); where at the end of each set, participants indicated their RPE using the FCR10. In a novel way, accuracy in the estimation of RIR was based on MPV and not on the number of repetitions, as this could increase subjectivity by having previously performed a set to failure (or if a set had continued until failure). In addition, this was supported by the greater variability in repetitions to failure (Rodríguez-Rosell et al., 2020) than when measuring MPV (Sánchez-Medina et al., 2017) in the back squat exercise at a moderate load. Specifically, accuracy was assessed by the difference (RIR_Dif) between the MPV of the RIR target (measured at visit 1) and the self-regulated RIR (measured at visit 2), not by the number of repetitions performed in the protocol until failure at the first visit, which was used to generate the individual RIR-velocity profile. For instance, if the participant completed the first self-regulated set (i.e., RIR3) at a MPV corresponding to RIR4 (i.e., 4 reps to failure), the final accuracy value was −1 (3 − 4 = −1). If MPV at RIR3 or RIR1 set did not correspond to any MPV specific value of the individual velocity-RIR profile, the MPV of the closest RIR associated with the autoregulation sets was considered. For instance, if the participant finished his/her set at 0.35 m•s^−1^, where RIR2 (i.e., 2 reps to failure) corresponded to 0.36 m•s^−1^ and RIR1 corresponded to 0.31 m•s^−1^ according to his/her velocity-RIR profile, the RIR considered was RIR2. All participants had 100% attendance to the protocols. The methodological design is shown in Figure 1.

### Statistical Analysis

Data are expressed as mean (M) and standard deviation (SD). Data normality was assessed using the Shapiro-Wilk test (*p* > 0.05). Parametric variables such as descriptive characteristics, MPV, VL, the RPE, and total of repetitions to muscle failure were compared between groups using the Student's *t*-test for independent samples. For the variables with variances that were not equal (months of experience and total reps in RIR3; Levene's test with *p* < 0.05) the Welch's T-test was applied, and for the non-parametric variables (total of reps in RIR1) the Mann-Whitney U-test was used, with the level of significance at *p* < 0.05. Effect size was calculated using Cohen's *d*, considering 0.20, 0.50, and 0.80 as small, medium, and large effects, respectively (Cohen, 1988); for total repetitions in RIR1 a biserial rank correlation was applied, considering 0.10, 0.30, and 0.5, respectively (Cohen, 1988). All analyses were performed with Jamovi® software (The jamovi project (2024); jamovi (Version 2.5) [Computer Software]. retrieved from https://www.jamovi.org; accessed on: 24 March 2024).

## Results

Overall, the groups showed similar characteristics. There were only significant differences in age and months of experience in RT (*p* < 0.05; *d* ≥ 1.66). The comparison between groups is shown in Table 1.

**Table 1:** Descriptive characteristics of each group.

	EG (N = 8) M ± SD	NG (N = 8) M ± SD	*p-value*	*d*
Age (years)	27.88 ± 4.12	20.75 ± 2.49	<0.001	2.09
Body mass (kg)	84.39 ± 13.99	70.80 ± 13.27	0.066	1.00
Body height (cm)	173.63 ± 9.02	170.38 ± 8.88	0.480	0.36
BMI (kg/m^2^)	27.85 ± 3.23	24.35 ± 4.17	0.082	0.94
Fat mass (%)	28.23 ± 10.73	22.84 ± 8.25	0.279	0.56
Muscle mass (%)	34.11 ± 6.74	37.54 ± 6.68	0.325	−0.51
Months of experience in RT	70.00 ± 52.27	8.38 ± 3.85	0.012^a^	1.66
1RM (kg)	139.80 ± 25.74	110.33 ± 31.49	0.060	1.02

EG = Experienced Group; NG = Novice Group; 1RM = 1 repetition maximum in back squat exercise on the Smith machine; d = Cohen's d effect size; ^a^ = Welch's T-test

No significant differences were found between the groups in the set to momentary muscular failure in both the MPV at RIR3 and RIR1 target (*p* = 0.380, *d* = 0.45; *p* = 0.349, *d* = 0.48, respectively), MPV to failure (*p* = 0.968, *d* = −0.02), VL from the fastest repetition (i.e., first or second rep; *p* = 0.968, *d* = 0.02), the RPE to failure (*p* = 0.418, *d* = 0.42), and total repetitions to failure (*p* = 0.601, *d* = 0.27). The results are shown in Table 2.

**Table 2:** Comparison of the momentary muscle failure set in the back squat at 70% 1RM between groups.

	EG (N = 8) M ± SD	NG (N = 8) M ± SD	*p-value*	*d*
MPV_RIR3_Target (m•s^−1^)	0.38 ± 0.07	0.35 ± 0.06	0.380	0.45
MPV_RIR1_Target (m•s^−1^)	0.32 ± 0.08	0.28 ± 0.05	0.349	0.48
VPM_Fail (m•s⁻^1^)	0.26 ± 0.07	0.26 ± 0.05	0.968	−0.02
VL_Fail (%)	49.60 ± 11.85	49.38 ± 9.78	0.968	0.02
RPE_Fail (0–10)	8.25 ± 1.16	7.63 ± 1.77	0.418	0.42
Total_Reps_Fail (No)	14.63 ± 6.07	13.38 ± 2.62	0.601	0.27

EG = Experienced Group; NG = Novice Group; VPM_RIR3_Target = mean propulsive velocity at 3 repetitions in reserve target before momentary muscle failure; VPM_RIR1_Target = mean propulsive velocity at 1 repetition in reserve target before momentary muscle failure; VPM_Fail = mean propulsive velocity to momentary muscle failure; VL_Fail = velocity loss from fastest repetition to momentary muscle failure; RPE_fail = rating of perceived exertion to momentary muscle failure; Total_Reps_Fail = total repetitions until momentary muscle failure; d = Cohen's d effect size

No significant differences were found between groups in both the MPV at RIR3 and self-regulated RIR1 (*p* = 0.673, *d* = 0.22; *p* = 0.891, *d* = −0.07, respectively), the RIR3_dif and RIR1_dif based on the target and self-regulated MPV (*p* = 0.955, *d* = 0.03; *p* = 0.717, *d* = 0.18, respectively), the VL from the fastest repetition (i.e., first or second rep) at RIR3 and self-regulated RIR1 (*p* = 0.708, *d* = 0.19; *p* = 0.097, *d* = 0.89, respectively), the RPE at RIR3 and self-regulated RIR1 (*p* = 0.172, *d* = 0.72; *p* = 0.169, *d* = 0.73), and total repetitions at RIR3 and self-regulated RIR1 (*p* = 0.560, *d* = 0.30; *p* = 0.914, *r* = 0.05, respectively). The results of RIR3 and RIR1 are shown in the top and bottom panels of Table 3, respectively.

**Table 3:** Comparison of repetitions in reserve sets in the back squat at 70% 1RM between groups.

	EG (N = 8) M ± SD	NG (N = 8) M ± SD	*p-value*	*d*
MPV_RIR3_SR (m•s^−1^)	0.40 ± 0.08	0.38 ± 0.07	0.673	0.22
RIR3_Dif	−1.19 ± 1.93	−1.25 ± 2.41	0.955	0.03
VL_RIR3 (%)	23.07 ± 8.83	21.19 ± 10.67	0.708	0.19
RPE_RIR3 (0–10)	5.75 ± 1.67	4.75 ± 1.04	0.172	0.72
Total_Reps_RIR3 (No)	10.63 ± 6.52	9.13 ± 2.59	0.560^a^	0.30
MPV_RIR1_SR (m•s^−1^)	0.33 ± 0.10	0.34 ± 0.07	0.891	−0.07
RIR1_Dif	−1.00 ± 1.98	−1.31 ± 1.33	0.717	0.18
VL_RIR1 (%)	35.95 ± 11.94	25.70 ± 11.11	0.097	0.89
RPE_RIR1 (0–10)	8.13 ± 1.25	7.25 ± 1.16	0.169	0.73
Total_Reps_RIR1 (No)	11.63 ± 6.05	10.13 ± 2.75	0.914^b^	0.05^c^

EG = Experienced Group; NG = Novice Group; VPM_RIR3_SR = mean propulsive velocity at 3 repetitions in reserve self-regulated; RIR3_Dif = Difference between target (visit 1) and self-regulated (visit 2) RIR3 based on mean propulsive velocity; VL_RIR3 = velocity loss from the fastest repetition to the self-regulated RIR3; RPE_RIR3 = rating of perceived exertion to the self-regulated RIR3; Total_Reps_RIR3 = total repetitions up to the self-regulated RIR3; VPM_RIR1_SR = mean propulsive velocity at self-regulated 1 repetition in reserve; RIR1_Dif = Difference between target (visit 1) and self-regulated (visit 2) RIR1 based on mean propulsive velocity; VL_RIR3 = velocity loss from the fastest repetition to the self-regulated RIR1; RPE_RIR1 = rating of perceived exertion to the self-regulated RIR1; Total_Reps_RIR1 = total repetitions up to self-regulated RIR1; ^a^ = Welch's T-test; ^b^ = Mann-Whitney U-test; ^c^ = Biserial rank correlation (r); d = Cohen's d effect size

## Discussion

The main finding of the present study is that experienced and novice RT subjects had similar objective accuracy in estimating RIR in the back squat exercise. Additionally, both MPV and the RPE were adjusted to the level of effort, regardless of the level of experience of participants. To the best of our knowledge, this is the first study to objectively compare the accuracy of RIR in groups with different levels of experience using valid (i.e., MPV) and simple (i.e., RPE) tools from an ecological approach to strength exercise. Finally, although we urge against generalizing the findings due to the sample size, given that the effect sizes were low to moderate in most of the variables compared to objectively estimate accuracy in RIRs, these results suggest that in practical terms both experienced and novice RT subjects have similar accuracy when estimating RIRs.

### RIR Accuracy According to the RT Experience Level

In practice, the use of RIR is a tool to implement self-regulation in RT programs, with the goal of achieving the desired level of stress or effort (Ormsbee et al., 2019). Recent evidence highlights that RT experience requires further research to clarify its influence on the RIR prediction (Bastos et al., 2024). However, RIR estimation studies assess this variable by the difference between the participants' prediction, either before (Steele et al., 2017) or during the set (Refalo et al., 2024; Remmert et al., 2023), with the total number of repetitions completed to muscle failure, which may affect the results. Similarly to the present study, Remmert et al. (2023) reported that training experience was not related to accuracy in estimating RIR5 to RIR0 in a set to muscle failure in the biceps curl, triceps extension, and seated row exercise at 72.5% 1RM in a mixed group of young adults; however, accuracy was measured by reported RIR during the set and repetitions to muscle failure in the same set, which may have been influenced by participants' intention to confirm their predictions. Likewise, Refalo et al. (2024) did not identify any relationship among RIR accuracy and biological sex, years of RT experience, and relative strength in the bench press exercise in a highly experienced RT sample (>7 years) of 24 men and women aged 18 to 40, highlighting the probability that the exercise performed may influence the accuracy of RIR predictions (Refalo et al., 2024). In this regard, a recent review by Halperin et al. (2022) pointed out that the accuracy for estimating RIR was lower in lower limb exercises, although the comparison between models suggested that the difference was not clear. Even though the present study did not compare upper and lower limb exercises, the results indicated that both experienced and novice subjects were able to similarly predict RIR with high accuracy in a traditional lower limb exercise. Other research indicates that college-age subjects with RT experience might be more accurate in estimating RIR as they demonstrate greater ability to perform a true 1RM lift (Zourdos et al., 2016), suggesting that there is likely a learning curve in novice subjects (Ormsbee et al., 2019; Steele et al., 2017). However, and in line with the above mentioned, this discrepancy could be explained by methodological differences in the assessment of accuracy. For instance, Steele et al. (2017) asked a group of 141 adults classified into five RT experience categories to predict the repetitions they could perform before starting the set and self-selecting the load to muscle failure. While those authors suggested a tendency towards improved accuracy with greater experience, we believe that they introduced subjectivity into the measurement of accuracy. In addition, it has been reported that experienced subjects with greater exposure to high efforts of low volume (few repetitions) are more accurate when reporting their level of effort (Testa et al., 2012), which could explain their greater ability to perform a true 1RM according to the effort applied (Zourdos et al., 2016). Similarly, Robinson et al. (2024) highlighted the psychophysiological contributions of greater exposure to the subjective experience of performing sessions near or to muscle failure, which could improve accuracy when reporting RIR. Therefore, the methodology employed in our study could have favored the participants accuracy by previously exposing them to a set to muscle failure in the same exercise in which they would be evaluated. In this sense, Remmert et al. (2023) noted that experience in training to muscle failure might have a greater influence on accuracy in reporting RIR than the RT experience in general. Thus, it is likely that familiarity with the protocol and exposure to a high or the maximal level of effort have a more determinant impact on accuracy in estimating RIR than RT experience accumulated over time. Although our results cannot be extended to a wide range of intensities, both groups reached a similar level of effort in relation to the MPV and the RPE of the set to failure, which implies the same ability to perform a maximal effort at moderate loads. In this sense, the RPE values < 9 could respond to the ecological approach applied to exercise, because when maximal intended velocity is requested in the concentric phase up to 100% of repetitions, the RPE values are usually higher (Varela-Olalla et al., 2022).

### Objective Support for Accuracy in Estimating RIR

Previous studies have reported that individualized load-velocity/RIR/RPE relationships provide a more accurate estimate of the relative load than generalized load-velocity relationships in experienced powerlifters (Balsalobre-Fernández et al., 2021b) which could be extended to the level of effort applied. In this sense, a strength of the present study is that the accuracy of the RIR estimation was supported by the individualized MPV and the RPE, allowing to reduce subjectivity in self-regulation when comparing subjects with different levels of experience. Recently, Mansfield et al. (2023) evaluated the reliability of mean velocity at RIR 3 and 0 through four sets to muscle failure in recreationally resistance trained males, finding no reliability in the bench press and prone rowing exercise. In contrast to our study, those authors analyzed the mean velocity at loads associated with 60 and 80% 1RM performing the concentric phase as fast as possible. Therefore, the lack of reliability may be related to the braking or deceleration phase of the bar at the top of the lift to maintain balance, suggesting the use of MPV to avoid underestimations in the neuromuscular potential since this braking phase occurs with loads up to ~76% 1RM (Banyard et al., 2018; Sanchez-Medina et al., 2010) as well as an individualized approach to RIR and the RPE (Balsalobre-Fernández et al., 2021b). In addition, Mansfield et al. (2023) mention that a possible reason why their results contrasted with previous research was that participants exercised with free weights, which allowed a mediolateral and anteroposterior movement that is not possible with a Smith machine. On the other hand, Morán- Navarro et al. (2019) indicated that MPV could estimate with high accuracy the proximity of muscle failure with loads of 65, 75 and 85% 1RM at maximal intended velocity in traditional exercises in young healthy men and, therefore, quantify more objectively the level of effort independently of the level of tested subjects. That study, despite not applying an ecological approach in the execution of the exercises and not requesting RIR from participants, supports the idea of objectification of RIR by MPV in subjects of different levels of experience (Morán-Navarro et al., 2019), which is in line with our results. Although we do not know how the application of maximal intended velocity might have affected the accuracy of the RIR in the present study, we believe that adopting an ecological approach as it has been previously proposed (Odgers et al., 2021), could positively influence accuracy because participants performed the intervention with the same technique (i.e., own speed) that they usually used in their RT.

RT performed to momentary failure generates high RPE and discomfort/pain values (Santos et al., 2021), which could lead to underestimate RIR when performing sets close to muscle failure (Armes et al., 2020), particularly, in subjects with poor handling of these tools. However, our results indicated that the RPE was linked to the level of effort applied, showing no differences between subjects of different levels of experience (i.e., EG vs. NG); although we are aware that these results should be taken with caution since RIR >3 was not analyzed as in other studies, despite the fact that these had another perspective in their analysis (Odgers et al., 2021; Zourdos et al., 2016). The RPE is also related to VL and the percentage or the number of repetitions in the upper limb exercise (Ormsbee et al., 2019; Varela-Olalla et al., 2022), generating a more comprehensive approach to the level of effort applied during RT, highlighting the RPE as a practical and simple tool to prescribe, control and quantify training by supporting RIR. In this sense, our research extends this notion, confirming the link among the RPE, VL, and the number of repetitions in a lower limb exercise, such as the back squat on a Smith machine. Nevertheless, it is important to note that our research focused on the accuracy of RIRs 1 and 3 in a single set of back squats; however, this is not typically the approach used in a RT program. Given that the length of the rest interval between sets affects mechanical performance and repetition volume (Janicijevic et al., 2025), we suggest caution when applying our approach.

In particular, our main findings may differ from those presented in other studies. For instance, Hernández-Belmonte et al. (2022) recently reported that in healthy men reaching RIR3 with loads of 70%1RM in a full squat, VL was ~40% in subjects of a low to a moderate relative strength level, whereas in our protocol, VL at the same effort level (i.e., RIR3) was ~23% and ~21% in the EG and NG groups, respectively (Table 3, top panel), but unlike in that study, participants stopped the set when they reached the self-regulated RIR. These differences could be linked to the fact that in the study by Hernández-Belmonte et al. (2022) maximal intended velocity was applied in the concentric phase, compared to the self-regulated speed proposed in the present study. In turn, Varela-Olalla et al. (2022) also using maximum possible velocity, showed that physically active men performing a set up to the maximum number of repetitions or close to it caused VL >60% in the bench press exercise, being higher than the VL <40% found by us in the RIR1 self-regulated set (Table 3, bottom panel), but in the back squat exercise. Therefore, the imposed movement velocity is a key factor affecting the level of effort, which may determine the discrepancies between the outcomes of different studies. However, in addition to being a simple and practical tool, the RPE could be more effective than VL to assess the level of effort or RIR when maximal intended velocity is not applied, since it has been shown to be a better predictor of muscle fatigue and the percentage or the number of repetitions than VL in the back squat exercise when subjects cannot and/or are not required to apply the concentric phase in an explosive manner (Zhao et al., 2023). While we are aware that performing the concentric phase at maximal intended velocity can maximize RT benefits in both upper and lower limbs (González-Badillo et al., 2014; Pareja-Blanco et al., 2014), the above information is relevant for scenarios such as rehabilitation or in untrained subjects, with our findings supporting the use of the RPE as a simple and practical tool to assess the level of effort independently of the subjects' experience. In addition to controlled execution speed, other variables such as the eccentric load and range of motion can be manipulated in RT according to the objective being pursued. In this sense, Suchomel et al. (2024) reported that greater braking impulses were produced during back squats with accentuated eccentric loading compared to traditional squats, without affecting movement velocity. Tsoukos et al. (2024) found that the range of motion of an exercise affected RT performance, particularly the number of repetitions that could be performed in a set. Unfortunately, those authors did not analyze how these training strategies might affect the accuracy of RIR estimation. Given that accentuated eccentric loading and range-of-motion manipulation are resources used in RT, future studies should examine how these strategies might affect the objective accuracy of RIR.

## Practical Implications

According to previous evidence (Hackett et al., 2017; Halperin et al., 2022; Refalo et al., 2024; Remmert et al., 2023) and the findings of the present study, training programs employing RIR as a self-regulatory tool can be effectively implemented in both novice and more advanced in RT subjects, where RIR are objectively supported by valid (i.e., MPV) and simple (i.e., RPE) tools to regulate the effort level. In this sense, we suggest prior familiarization with high to maximal levels of effort (i.e., testing to momentary muscle failure) improves the accuracy of subsequent RIR estimates, something that can be linked to a learning effect as proposed in previous studies, although from a different perspective (Remmert et al., 2023). Therefore, incorporating introductory sessions that teach participants to self-regulate their level of effort related to RIR could be beneficial to optimize training and the expected effects (Hernández-Belmonte et al., 2022; Pareja-Blanco et al., 2017, 2020). In addition, our results and those of other studies (Bachero-Mena et al., 2025; Balsalobre-Fernández et al., 2021b) support an individualized approach to the level of effort; therefore, we encourage professionals to consider this approach.

## Limitations and Future Studies

The main limitation of the present study is the sample size, particularly that of women, which prevents generalization of our findings even though they may be relevant to practice. Future studies should apply the approach presented here to consolidate or refute the results. Additionally, another limitation is that in our study, we focused exclusively on the Smith back squat exercise at moderate intensity (i.e., 70%1RM) and RIR 1 and 3 in subjects aged 18 to 34 years. This does not allow generalizing the results to other exercises, external loads, RIR, or ages. Future research should analyze other exercises (e.g., bench press), external loads, and RIR, and include a larger number of women, in order to explore possible differences. In addition, it might be interesting to compare bilateral vs. unilateral and multi-joint vs. single-joint exercises, as well as RT programs incorporating RIR alongside the RPE (and if possible MPV) as a control of the level of effort in subjects with different levels of experience. Also, it could be relevant to study previous experience in the use of the RPE and RIR tools in order to analyze the effect of experience in the estimation of RIR not only in terms of RT, but also for self-regulation of the load. Finally, assessing affective valence in RT is an emerging strategy to assess the level of effort based on the percentage or the number of repetitions (Emanuel et al., 2021), thus it could be attractive to analyze its relationship with RIR and the RPE to improve accuracy and objectivity in effort self-regulation by means of practical and simple tools.

## Conclusions

In conclusion, RIR are an objective tool for self-regulation of the level of effort in the back squat exercise, regardless of RT experience. In addition, familiarity with the training methodology and different levels of effort might be more influential on the accuracy of effort estimation than experience itself. The RPE emerges as a simple and practical tool to support the objective use of RIR and regulate the level of effort in the back squat exercise when MPV is not accessible, with the purpose of optimizing the RT prescription and effects in general populations.
